# Supplementary material for: Speeding up quantum perceptron via shortcuts to adiabaticity
Source: Sci Rep. 2021 Mar 11;11:5783. doi: 10.1038/s41598-021-85208-3 (PMC7952456; doi:10.1038/s41598-021-85208-3)
Supplement: Supplementary file 1 — Supplementary material 1 (pdf 1116 KB) [file 41598_2021_85208_MOESM1_ESM.pdf]

Supplementary Information for "Speeding up quantum perceptron via shortcuts to adiabaticity"

Yue Ban,[1,2,*] Xi Chen,[1,3,†] E. Torrontegui,[4,5,‡] E. Solano,[1,3,6,7,§] and J. Casanova[1,6,¶]

[1] *Department of Physical Chemistry, University of the Basque Country UPV/EHU, Apartado 644, 48080 Bilbao, Spain*
[2] *School of Materials Science and Engineering, Shanghai University, 200444 Shanghai, China*
[3] *International Center of Quantum Artificial Intelligence for Science and Technology (QuArtist) and Department of Physics, Shanghai University, 200444 Shanghai, China*
[4] *Departamento de Física, Universidad Carlos III de Madrid, Avda. de la Universidad 30, 28911 Leganés (Madrid), Spain*
[5] *Instituto de Física Fundamental IFF-CSIC, Calle Serrano 113, 28006 Madrid, Spain*
[6] *IKERBASQUE, Basque Foundation for Science, Maria Diaz de Haro 3, 48013 Bilbao, Spain*
[7] *IQM, Munich, Germany*

QUASI-OPTIMAL-TIME SOLUTION BY INVERSE ENGINEERING

In the main text, we have introduced the inverse engineering (IE) to find the control field and obtain the sigmoid transfer function. Here, we provide the detailed comparison of transfer functions and driving fields between IE and FAQUAD methods for the operation time $t_f = 0.3$, see Fig. S1. The transfer functions for both IE in the case of $\theta = \sum_{i=0}^{3} a_i t^i$ and FAQUAD protocols can reach 1 and 0 at $x_j/\Omega_f = x^{\max}$ and $x_j/\Omega_f = -x^{\max}$ ($x^{\max} = 12$) with high fidelity, respectively. However, the driving field $\Omega(t)$ for IE decreases more smoothly from the maximum value $\Omega(0) = 1999.5$ for $\kappa = 2000$, which makes the experimental implementation more feasible.

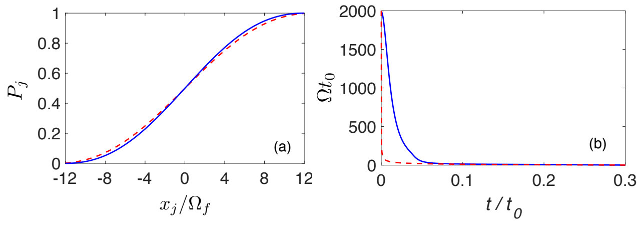

FIG. S1. With $t_f = 0.3$, we present the transfer function (a) and the external field $\Omega(t)$ (b) obtained from IE with $\theta = \sum_{i=0}^{3} a_i t^i$ (solid-blue) and FAQUAD (dashed-red). In both cases, $\Omega(t)$ is designed when $y/\Omega_f = 12$.

We clarify the manner of doing quasi-optimal-time control as follows. The coefficients of the polar angle $\theta = \sum_{i=0}^{s} a_i t^i$ with $s = 3$ can be solved from the boundary conditions of $\theta(0)$, $\theta(t_f)$, $\dot{\theta}(0)$, $\dot{\theta}(t_f)$ for a fixed value t_f. The polar angle can also be set into a higher order polynomial ansatz ($s > 3$), where the unknown free coefficients can be scanned to seek for a lowest C value.

	N order	$P(-x^{\max})$	$P(x^{\max})$	C
Polynomial	3	0.204	0.998	0.206
	4	0.024	0.998	0.026
	5	0.0065	0.998	0.008
Trigonometric	2	0.219	0.998	0.221
	3	0.0534	0.998	0.0554
	4	0.0429	0.998	0.0389
Exponential		0.086	0.998	0.0878
FAQUAD		0.204	0.796	0.41

TABLE I. Comparison of the performance of different ansatz: polynomial, trigonometric and exponential functions in form introduced in the main text, with $t_f = 0.15$, $x^{\max} = 12$, $y/\Omega_f = 12$.

For $t_f = 0.15$, we first set $s = 4$, and obtain $a_0 = \theta(0)$, $a_1 = \dot{\theta}(0)$, a_3 and a_4 and the functions of a_2 by fixing the boundary conditions Eq. (8equation.0.8) and Eq. (9equation.0.9) in the main text. As shown in Fig. S2, the minimum $C = 0.026$ can be found at $a_2 = -391$. By using the same boundary conditions, we set $s = 5$, a higher order polynomial ansatz, where $a_0 = \theta(0)$, $a_1 = \dot{\theta}(0)$, a_4 and a_5 are the functions of a_2 and a_3. The relation of C value versus a_2 and a_3 are demonstrated in Fig. S3, where the range of $C < 0.01$ manifests itself as a stripe area. We find numerically C value reaches its minimum at 0.0087 when $a_2 = -50$ and $a_3 = -3980$.

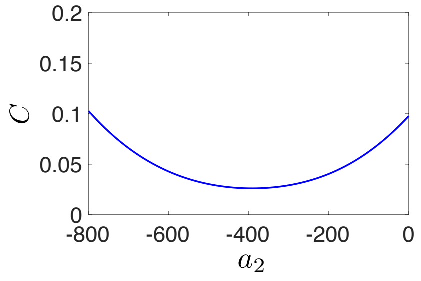

FIG. S2. With $t_f = 0.15$, the dependence of C value on the free parameter a_2, where $\theta = \sum_{i=0}^{4} a_i t^i$, and $y/\Omega_f = 12$.

---

* ybanxc@gmail.com
† xchen@shu.edu.cn
‡ eriktorrontegui@gmail.com
§ enr.solano@gmail.com
¶ jcasanovamar@gmail.com

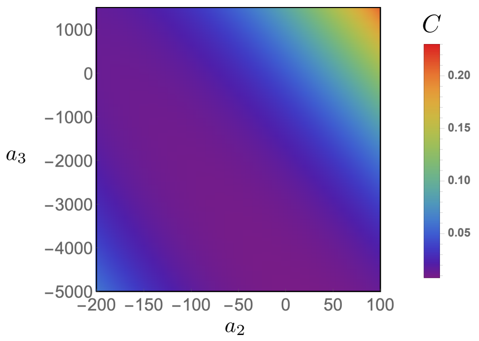

FIG. S3. With $t_f = 0.15$, the dependence of the density contour plot of C on the free parameters a_2 and a_3, where $\theta = \sum_{i=0}^{5} a_i t^i$ and $y/\Omega_f = 12$.

Using the same strategy to search for a minimal C value for a fixed value t_f, we demonstrate C value in the function of t_f, as shown in Fig. 5 (a) Dependence of C as a function of the final time t_f, using IE in the cases of $\theta = \sum_{i=0}^{3} a_i t^i$ (solid-blue), $\theta = \sum_{i=0}^{5} a_i t^i$ (dotted-black) and FAQUAD (dashed-red). The inset of (a) shows the corresponding transfer functions for $t_f = 0.15$, where the dotted-black curve represents the quasi-optimal-time solution with $a_2 = -50$, $a_3 = -3980$. (b) For $t_f = 0.15$, the driving field $\Omega(t)$ designed from IE in the cases of using $\theta = \sum_{i=0}^{3} a_i t^i$ (solid-blue), using $\theta = \sum_{i=0}^{5} a_i t^i$ with the optimal parameters $a_2 = -50$, $a_3 = -3980$ (dotted-black), and $y/\Omega_f = 12$. figure.caption.5 (a) of the main text, where the minimal operation time t_f reaches at $t_f^{\min} = 0.15$ for $C < 0.01$. Numerical calculations prove that further setting higher order of polynomial ansatz ($s > 5$) does not improve to shorten $t_f^{\min}$.

The detailed comparison between STA and optimal control theory is presented in Ref. [1], proving that IE method allows to approach the performance gained from optimal control theory by introducing more freedom in polynomial or trigonometric ansatz of θ. Here, we present the comparison of the performance of activation function by using IE with polynomial function $\theta = \sum_{i=0}^{N} a_i t^i$, trigonometric function $\theta = a_0 + a_1 t + \sum_{i=2}^{N} a_i \sin[(i-1)\pi t/t_f]$ and exponential functions $\theta = a_0 e^t + a_1 e^{-t} + a_2 e^{mt} + a_3 e^{-mt}$ with $m = 25$ as well as FAQUAD, shown in Table I, showing that higher polynomial ansatz gives a quasi-optimal-time solution.

[1] Martikyan, V., Guéry-Odelin, D. & Sugny, D. Comparison between optimal control and shortcut to adiabaticity protocols in a linear control system. *Phys. Rev. A* **101**, 013423 (2020).
